# Acute Thyroidism Following Curettage

**Published:** 1904-07-23

**Authors:** 


### ACUTE THYROIDISM FOLLOWING CURETTAGE.

Wells[1] describes a case of acute thyroidism following currettage for endometritis. The right lobe of the thyroid was enlarged and the patient was a nervous thin woman with a rapid pulse. There was no protrusion of the eyeballs. She did not take ether well, and six hours after the operation she was flushed and voluble but not worried, and her mind was clear; her pulse had risen to 130 and became more rapid with excitement. The condition got worse and lasted for 23 days, not responding to treatment, and then subsided.

[1] Med. News, June 25, 1904.

